# Otorrhœa*Read before the Buffalo Medical Club, Sept. 14th, by Dr. F. W. Abbott, and requested for publication by the Medical Journal Association.

**Published:** 1881-10

**Authors:** F. W. Abbott


### BUFFALO

### Medical and Surgical Journal.

Vol. XXI. OCTOBER, 1881. No. 3.

### Original Communications.

### Otorrhœa.*

*Gentlemen of the Medical Club*—A few days ago I received a note from the gentleman whose turn it is to read a paper to-night, in which he asked me to supply his place as he could not be here. I consented and have done what I could in the very brief time which I have had to spare. I have had no time to read upon the subject and can give you only a few practical points which experience has taught me.

In this familiar paper I shall not attempt to be logical, consistent or exhaustive, but shall consider the various topics in the order in which they happen to occur to me, using the English nomenclature by preference, but introducing the Latin whenever it will conduce to brevity, or euphony.

Otorrhœa means a running from the ear. It is a broad term and includes every appearance of fluid at the external auditory meatus. We will exclude, however, traumatic hæmorrhages as not coming within the category of true otorrhœas. The ordinary otorrhœas are purulent and muco-purulent, and these occasionally tinged with blood. As these discharges are always a sign of inflammatory action somewhere in the ear, whenever

* Read before the Buffalo Medical Club, Sept. 14th, by Dr. F. W. Abbott, and requested for publication by the Medical Journal Association.

they occur, we have an otitis either acute or chronic, and these may be located either in the external ear, giving us an otitis externa, acute or chronic, or in the middle ear, giving us an otitis media, acute or chronic. There are a few complications of these different forms of otitis which so frequently occur that they have almost attained the dignity of separate diseases in our nomenclature. Among these the secretion of pus is the most common perhaps, and this gives us purulent otitis externa, acute or chronic, and purulent otitis media, acute or chronic. With the chronic form of purulent inflammations polypi are often developed and the presence of these should be indicated in a careful diagnosis, and so we have as a common name chronic purulent otitis externa, or media, with polypi.

Some go so far as to mention the fact of a perforation of the tympanic membrane with chronic purulent inflammations of the middle ear, and give us chronic purulent otitis media with perforation, but perhaps this is an extra refinement, for if there is a formation of pus in the middle ear, it must, if it appears externally, come through a perforation in the drum membrane. Inflammations of the external ear may be either circumscribed or diffuse, giving us acute circumscribed otitis externa, acute diffuse otitis externa, chronic circumscribed otitis externa and chronic diffuse otitis externa. Any of these forms may produce a true otorrhœa, although it is temporary in the acute cases.

From this long list of different diseases, which are all accompanied by an aural discharge, we see at once the necessity of a careful inspection of each case, to learn the exact source of the discharge, before attempting to treat it.

In considering these affections in detail, we will commence with the external meatus and proceed inwards. The most common otorrhœas having their origin in the external meatus should be traumatic, being produced by the surgeon's knife. Circumscribed inflammations, alias boils, are very frequent in this locality, and the proper treatment for these is an early and free incision. If the boil is near the concha, reflected light is not absolutely necessary, but if it is some ways within, direct illumination is unsatisfactory. When you see the point you want to cut, introduce a narrow-bladed knife, with its back to the wall of the meatus, and pierce the swelling and cut towards the centre. Of course, your patient will jump, but let him; you must pierce as deep as you want to go before he has time to jump, and as he gets away from you the knife cuts its way out of the apex of the boil. If you cut down onto it, your knife is apt to keep on in the same direction after it leaves the meatus and you gash the tragus or anti-tragus, and your patient objects to a slashed ear. If you produce a satisfactory otorrhœa by this means, cleanliness by means of warm water and a syringe completes the cure. A free and early incision is considered to be a good prophylactic against the recurrence of these boils, which is often one of the most troublesome features of these cases. Should they recur, however, sulphide of colcium in one-tenth grain doses, every four hours, is the thing to give. I prescribe it frequently and am satisfied empirically that it does have an effect in preventing recurrent boils.

There is a form of genuine otorrhœa dependent upon diffuse inflammation of the external meatus, which is sometimes quite profuse, and if its true source is not recognized and correctly treated, may run on for years. This inflammation is eczematous in character, and fostered by the warmth and moisture of its habits, and encouraged by various wet applications, and especially by the application of glycerine, which to some minds appears to be a specific for all kinds of ear trouble, it runs on and the ear itches and burns until life becomes a torment. The first thing to do with such an ear is to cleanse it thoroughly with warm water and syringe, and dry it with absorbent cotton. The whole meatus, from anti-tragus to membrane, is red and swollen with ridges, fissures and excoriations. The first direction to give is, that it must be kept perfectly dry. The surgeon must cleanse it with water, if necessary, and dry it as soon as possible, but the patient must not wet it at all. In my practice the best results have followed the use of a .10 or .15 solution of nitrate of silver, thoroughly applied after the skin is dry and clean, using a saturated wisp of absorbent cotton on a cotton holder. After a few minutes I dry it carefully again, and follow this up every day, if it gets moist in one day. Very soon the discharge decreases, so that no moisture appears, but the discharge dries in flakes as fast as secreted. Then, after applying the nitrate of silver, I sometimes apply a thin coating of yellow oxide of mercury ointment, made with cosmoline. Occasionally a polypus is found hanging to the wall of the external meatus with a perfect drum membrane, although they are rare; so I will say what I have to say of the treatment of polypi here. A profuse otorrhœa often comes from a little polypus, which may be nothing apparently but a small granulating surface, so this is one of the first things to look for in a running ear. When found, it isn't always necessary to scare your patient to death by telling him he has a polypus. If he is nervous and imaginative, he will picture a many-rooted monster sending claws deep into his brain, or ask anxiously if it is a cancer. But at all events get rid of your polypus. If it is pediculated, pick it off with forceps or snare and touch its point of attachment with chromic acid. If it has a broad base, cover it with chromic acid and repeat the application from time to time. It may be necessary to scrape the exuberent granulation with a curette. If there is denuded carious bone, it is necessary to scrape this well until you reach sound tissue.

But by far the most abundant source of the discharge in otorrhœas is the mucous membrane lining the cavity of the middle ear. An acute inflammation of the pharyngeal mucous membrane, especially such as accompanies scarlatina, and after this a long-ways measles or an idiopathic pharyngitis extends by contiguity of tissue through the Eustachian tube to the middle ear. Mucus is secreted and fills the cavity, the inflammation grows more severe, pus is formed, and, having no means of exit, finally breaks through the drum membrane and shows itself externally. But this bony cavity cannot empty itself completely through this orifice. Some pus stays, perhaps decomposes and becomes a source of irritation. More is secreted, which goes through the same processes, and finally we have lining the middle ear, a pyogenic membrane, and a continuous discharge. Complications soon ensue; the continued moisture and warmth encourage the growth of granulations, and polypi spring up. Perhaps the inflammation spreads to the mastoid cells, and here we have as a result ulceration, necrosis, finally an opening externally and relief from pain and fever with a sinus connecting the surface of the mastoid bone with the middle ear. Perhaps the ulceration and necrosis extend inwards, reach the meninges of the brain, setting up meningitis, acute, spreading, with death, or chronic meningitis with all its attendants, epilepsy, mania, dementia, etc. Perhaps ulceration begins in the middle ear; its cover is a thin lamella of bone, which separates it from the dura mater here. Necrosis often, sooner or later, destroys this lamella, and the dura mater becoming implicated, we have the same results of meningitis as mentioned before.

So a running ear is not simply a source of annoyance, as it is often considered, still less a sort of drainage tube, letting off foul humors, deleterious to the system, which must not be interfered with, as is still the belief in some quarters; but it is a sword of Damocles hanging continually over the head of its possessor and threatening life itself.

Since the results of a chronic purulent otitis media are or may be so appalling, the question of the proper treatment becomes a very urgent and important one, and it will not do for any physician into whose hands a family have entrusted themselves in good faith, relying upon his knowledge of disease, to say, as I have too often heard, "Well, I don't know anything about ear diseases, and I don't want to." This disease is usually very easily diagnosticated and the rationale of the treatment is very simple: cleanse the ear thoroughly with syringe and absorbent cotton, and inspect it through a speculum with reflected light. A speculum may be bought for fifty cents, and in these days every physician should have a laryngoscopic mirror, but if you haven't, take a bit of looking glass and scrape a little hole through the silver. With this simple reflector you can light up the meatus so that you can tell whether its walls look healthy or not, and if they do, you can reason by exclusion that the middle ear is affected, even though you do not get a satisfactory view of the drum membrane.

Usually a hole in the ear drum can be seen and distinguished, but often it cannot. It may be in the lower anterior segment, out of sight, around the bend in the external meatus; or the drum membrane may be inflamed and discolored so that you are not sure whether it is drum membrane which you see or the internal wall covered with mucous membrane. Sometimes you can diagnosticate a perforation by listening closely while the patient inflates the Eustachian tube and middle ear by the Valsalvian method. If a perforation exists and the Eustachian tube is pervious, you will hear the whistle of the air as it passes through. I have read somewhere of putting a light bit of cotton in the ear in such cases and expecting to see it blown out, but this experiment has never succeeded under my observation. If you cannot hear the whistle of air, and are still in doubt, drop a little warm water into the ear and watch it through the speculum while the patient inflates the ear, and you may be rewarded by seeing bubbles of air break through the water. Of course, if you have a Politzer bag you wont linger over the Valsalvian method, and one pull through a Siemen's speculum settles the case, but I am not supposing that you have an Otologist's armament.

Having established your diagnosis, the treatment comes next. You want to restore that mucous membrane to its normal condition, where it is possible, and induce a cicatrization of ulcerated surfaces. The first need is cleanliness, and cleanliness without violence. For this you will need more tools. A mirror on a head band, which leaves both hands free, is absolutely essential; no one has any business with a purulent otitis media without one. After syringing the ear long and carefully, dry it out with absorbent cotton, under your eye. You must always look and see where you are going, the structure of the ear is too delicate for blind poking. Patients and friends cannot cleanse an ear, you must do it yourself, or it won't be done. I know one man who after 15 or 20 years' practice can cleanse his own ear. He puts a piece of slender flexible rubber tubing on the nozzle of his syringe and inserts that into his ear; as he has no ear drum, the way is clear to the bottom of the hole. After pumping from a pint to a quart of water through this, he dries his ear with absorbent cotton on a cotton holder, and this he can insert clear into the middle ear. As he does this every day, no pus collects and hardens. This is the single exception that proves the rule that no man can cleanse his own ear. After the ear is clean, the question of what application to make comes up, and here a pretty wide choice presents itself, and the very variety of the remedies which have been mentioned only proves that no one has been found entirely satisfactory. I more frequently begin with blowing in finely-powdered alum than in any other way, and repeat this every other day. I often, when this fails or seems to have too little effect upon the inflamed membrane, use finely-powdered iodoform or iodoform and alum. The great objection to iodoform is its odor, and this becomes very disagreeable in using iodoform in this manner. When one puffs a cloud of iodoform into a patient's ear, the return blast loads his mustache with the powder and he carries it under his nose the rest of the day. Some Medical Journals, a few months ago, vaunted boracic acid as the application in purulent otitis media, but in my hands it has not proved a specific by any means, but a useful variation. These powders act better when the drum membrane is almost entirely destroyed, I think, and a puff applies them thoroughly to every part. When the perforation is small and in the upper part of the membrane, the case presents peculiar difficulties. It is very difficult to cleanse such an ear, and here especially, after syringing thoroughly, it is beneficial to blow through the Eustachian tube by Valsalva's method, or the air bag, to force the middle ear fluids through the perforation in the external meatus, where they can be wiped out with cotton. In such cases I have had the best results from the use of strong solutions of nitrate of silver .10 to .15. After cleansing the ear by all ways as thoroughly as possible, I have the patient lie on the side, with the affected ear uppermost. Then with a pipette introduced well into the ear, I drop in 10 to 15 drops. I then pull and work the ear so as to churn it down clear into the middle ear, and be sure that it reaches every part. After leaving it a few minutes, I draw out what I can with the pipette, and put in a little pure warm water, then pump this out and put in some more, finally I drop in a solution of salt to decompose the nitrate of silver that may be left. The principal object of this procedure is cosmetic. Should the patient arise with an ear full of a .15 solution of nitrate of silver, a long, dirty-brown streak from ear to shirt collar would confront you at the next visit. Sometimes you have the good fortune to see a perforation close up, which is a consummation most devoutly to be wished, as this protects the mucous membrane of the middle ear from external irritants. If the drum membrane does not close up and the secretion stops, it is best to wear a loose fledget of cotton for a protection.

Dr. Howe spoke very highly of permanganate of potash in solution, for purulent otitis media, in a paper published some time ago. He gave it to the patients for ear drops. I used it some, but the patients objected to the stains left by this salt on everything it touched, and in my hands it did not prove as efficacious as I had hoped it might from the statistics given in its favor.

Where patients cannot come to the physician for treatment, they should provide themselves with a good ear syringe and keep the ear as clean as possible. If all the pus and mucus is not removed, it can be kept fluid and wholesome, so that none will be blocked in behind dry hardened masses, and the penetrating odor, which makes so many running ears an offense to the whole household, will be prevented. Should mastoid-cell complications arise, an incision should be made into the mastoid at once with strong knife or trephine.

There is much more that might be said about otorrhœa, but I have occupied my half hour, and I don't think I ought to read any more to-night.

BUFFALO, Sept. 17th, 1881.

*Gentlemen*—My incomplete paper on otorrhœa, which you have kindly expressed a desire to publish, was supplemented, in some particulars, in the discussion upon it which followed its reading. Thinking that these questions might arise in the minds of some of your readers, I have taken the liberty of appending a few of them with their answers as well as I can remember them.

Dr. D. The doses of sulphide of calcium as given in the paper (1-10 grain) are entirely inert. Experiments have shown that 1-grain doses are required to produce any effect, and 5-grain doses may be given without ill effects.

Answer by Essayist. I have never failed in stopping recurrent boils in the ear by the use of 1-10-grain doses of this drug. When I do, I shall use larger doses, but shall recommend the smaller ones until they fail.

Ques. Is sulphide of calcium useful in boils occurring elsewhere than in the ear?

Dr. M. I was cured by it last spring of recurrent abscesses in different parts of the body.

Dr. G. I am disappointed that nothing was said in the paper about the treatment of the acute stages of otitis media, which occurs especially in the course of the exanthemata. Will you say something on this point.

Ans. This subject did not come strictly within the range of my subject, but I will say in all cases of acute otitis media I use first hot water, directing the patient to hold his head over a bowl and continually instilling into the ear water as hot as can be borne, keeping the supply hot by adding boiling water. This to be continued 10 to 15 minutes or until the pain ceases. Repeat this every hour or two, using in the meantime ear drops of Majendie's solution of morphine. If the pain is not entirely relieved by this, apply some leeches to anti-tragus and tragus, putting some cotton into the ear to prevent them from going in and fastening onto the drum membrane.

Dr. H. Is there any means of telling whether there is a collection of fluid in the drum cavity in these cases, and if so, what is the remedy?

Ans. Yes; upon inspection the drum membrane appears to bulge outwards, and it has lost its translucent look. Then an incision should be made through its lower posterior part.

Ques. Is there any danger that this incision will not heal up?

Ans. No. It is extremely difficult to maintain a traumatic perforation of the drum membrane in cases where it is desirable.

Dr. M. Is not such an application of nitrate of silver, as you describe, very painful?

Ans. It is not. It causes no pain, if the cuticle lining the external meatus is intact.

Ques. Is not the chromic-acid application painful?

Ans. It is not, if the application is made only upon the polypus, and the acid is not allowed to touch any healthy tissue. I twist a little point of cotton on my cotton holder and dip just the tip into some chromic acid, which has deliquesced and become fluid without the addition of any solvent; with this I touch just where I want to. If it should run onto sound tissue and cause pain, this can be relieved by gently syringing with warm water.

Yours truly,

F. W. ABBOTT, M. D.

*The Medical Journal Association.*

